# Modulating Anxiety‐Like Behaviors in Neuropathic Pain: Role of Anterior Cingulate Cortex Astrocytes Activation

**DOI:** 10.1111/cns.70227

**Published:** 2025-01-21

**Authors:** Qingqing Zhou, Qi Zhong, Zhuang Liu, Ziyue Zhao, Jie Wang, Zongze Zhang

**Affiliations:** ^1^ Department of Anesthesiology Zhongnan Hospital, Wuhan University Wuhan China; ^2^ Department of Neurology, Songjiang Research Institute, Shanghai Key Laboratory of Emotions and Affective Disorders Songjiang Hospital Affiliated to Shanghai Jiao Tong University School of Medicine Shanghai China; ^3^ Key Laboratory of Magnetic Resonance in Biological Systems, State Key Laboratory of Magnetic Resonance and Atomic and Molecular Physics, National Center for Magnetic Resonance in Wuhan Innovation Academy for Precision Measurement Science and Technology, Chinese Academy of Sciences Wuhan China; ^4^ University of Chinese Academy of Sciences Beijing China

**Keywords:** anterior cingulate cortex (ACC), astrocyte, chemogenetic approach, excitatory neurons, spared nerve injury

## Abstract

**Aims:**

The comorbidity of anxiety‐like symptoms in neuropathic pain (NP) is a significant yet often overlooked health concern. Anxiety sufferers may have a lower tolerance for pain, but which is difficult to treat. Accumulating evidence suggests a strong link between astrocytes and the manifestation of NP with concurrent anxiety‐like behaviors. And the anterior cingulate cortex (ACC) has emerged as a key player in pain modulation and related emotional processing. However, the complex mechanisms that astrocytes in ACC influence anxiety behavior in mouse models of NP remain largely unexplored.

**Methods:**

Utilizing the traditional spared nerve injury (SNI) surgical model, we employed chemogenetic approaches, immunofluorescence, and western blot to investigate the functional significance and interactive dynamics between ACC astrocytes and excitatory neurons.

**Results:**

Our results revealed that SNI surgery induces NP and delayed anxiety‐like behaviors, accompanied by increased astrocyte activity in the ACC. Chemogenetic manipulation demonstrated that inhibiting astrocytes alleviates anxiety symptoms, while activating them exacerbates anxiety‐like behaviors, affecting local excitatory neurons and synapse density. Direct manipulation of ACC excitatory neurons also significantly impacted anxiety‐like behaviors.

**Conclusion:**

Our results highlight the pivotal role of ACC astrocytes in modulating anxiety‐like behavior, suggesting a novel therapeutic strategy for anxiety associated with NP by targeting astrocyte function.

## Graphical abstract

Our finding elucidated the regulatory role of ACC astrocytes in neuropathic pain comorbid with anxiety behaviors. The modulatory effect of astrocytes on anxiety may occur by influencing the synaptic‐related proteins, which in turn affects the activity of excitatory neurons.

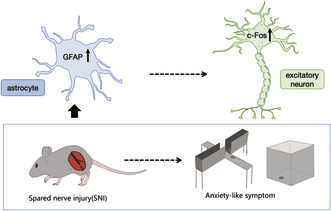

## 1 Introduction

Neuropathic pain (NP), which is caused by damage or disease of the somatosensory system, has been demonstrated to be highly comorbid with various psychiatry conditions, such as anxiety, depression, and posttraumatic stress disorder (PTSD) [1, 2, 3, 4]. Among these comorbidities, anxiety plays a significant role in intensifying pain perception and reducing pain tolerance, rendering it intractable and hard to treat. Despite the high comorbidity of anxiety among NP patients, more than half of comorbid anxious patients could not benefit from effective treatments, which highlights the urgent need for better therapeutic strategies [5, 6, 7]. Currently recommended first‐line anti‐pain (opioid analgesic) treatments for NP have obvious side effects that prolongs the duration of hyperalgesia and induce anxiety disorder [7, 8, 9]. This problematic feature of the current NP therapeutic strategies urges us to advance our understanding of the NP‐mediated anxiety mechanism.

Many brain regions, such as the anterior cingulate cortex (ACC), medial prefrontal cortex (mPFC), central amygdala (Ce), insular cortex (IC), and ventrolateral periaqueductal gray (vlPAG), contribute to the central mechanisms of NP [1, 10]. Among these regions, ACC is particularly notable for its role in anxiety regulation. For example, elevated activity was observed in this region during a mammal model with experimental anxiety, depression, and social deficit [11]. Furthermore, functional MRI and experimental evidence also verified that ACC exhibited hyperactivity in SNI mice with anxiety‐like behaviors [12, 13]. Optogenetic activation or inhibition of ACC pyramidal neurons could respectively induce or ameliorate anxiety‐like actions [8, 14, 15]. Stimulation and transcranial magnetic stimulation of ACC could serve as effective treatments of the affective component of chronic pain [16]. Furthermore, one research proposed that the presynaptic LTP in ACC neurons could be a key cellular mechanism for anxiety behavior caused by chronic pain [17]. Meanwhile, a recent study suggests a functional involvement of ACC PVINs in mediating the anxiety induced by maternal separation in mice [18]. These pieces of evidence suggest that the ACC plays a vital role in NP comorbid anxiety, serving as a critical target for understanding and potentially mitigating the emotional disturbances associated with NP.

Several former studies of NP were mainly focused on the variations of neurons or microglia, often paying insufficient attention to the function of astrocytes [19, 20]. Previous findings suggested that NP‐mediated development of negative emotions was triggered and maintained by changes in neuronal plasticity and neuronal firing activity [21, 22]. However, astrocytes are the most abundant glial cell type in the central nervous system (CNS), historically thought to primarily provide necessary support for neurons [23, 24]. Emerging evidence indicates that astrocytes in the CNS are involved in pain processing and emotional regulation. For instance, astrocyte ablation is sufficient to induce negative emotions and impair cognitive flexibility [25]. Reactivation of astrocytes in ACC contributes to allergic inflammation‐induced anxiety‐like behavior [26]. Furthermore, reduced level of GFAP in the hippocampus could be detected in anxious rats which ongoing PTSD [27]. By using astrocyte‐specific manipulation approaches in the central amygdala of mice and rats, researchers highlights astrocytes involve in modulation of emotional states under normal and chronic pain conditions [28]. These prior studies suggested that astrocyte may play a significant role in the development of anxiety behavior. However, studies on the modulating effects of astrocytes on anxiety have focused on other brain region and diseases. Besides, there are many studies reports that astrocytes should play a significant role in the initiation and maintenance of chronic pain through the release of key pro‐inflammatory cytokines [29, 30]. Some researches also find that low intensity transcranial direct current stimulation (tDCS), allodynia‐like behaviors of mice that performed partial sciatic nerve ligation (PSL) surgery can be reversed [31]. However, previous evidence in terms of ACC astrocytes mainly focused on remyelination and cognitive deficits [32], inflammatory pain [33], and lactic acid metabolism changes, followed by pain‐related aversive learning and decision‐making performance [34, 35]. Ambiguous results still exist regarding the putative mechanisms of how ACC astrocytes influence SNI mice anxiety behavior. This gap in research points to an underexplored area in understanding the role of astrocytes in NP and its comorbid emotional disorders.

To determine whether astrocytes in ACC may contribute to emotional disorders, we used the spared nerve injury (SNI) model, which is one of the most well‐validated NP models [36, 37]. By employing the designer receptor exclusively activated by designer drugs (DREADDs) approach, along with behavioral tests and immunohistochemical methods, our results highlight the critical involvement of astrocytes and astrocyte‐mediated activation of excitatory neurons in the ACC in NP comorbid anxiety, offering new avenues for therapeutic intervention targeting astrocyte function. The integration of these findings into the broader context of NP research underscores the necessity of a more comprehensive approach that includes the role of astrocytes in both pain and emotional regulation.

## 2 Materials and Methods

### 2.1 Animals

**FIGURE 1:**
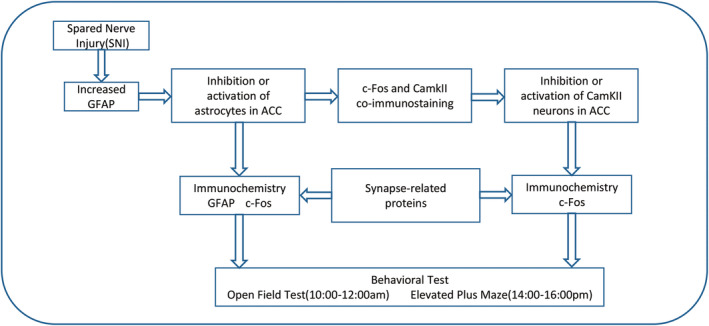
Flowchart of the experimental design.

Experiments were performed using nine‐week‐old male C57BL/6J mice (25–28 g) obtained from Liaoning Changsheng Biotechnology Co., Benxi, Liaoning, China. The mice were group‐housed with a maximum of five animals per cage and maintained under a controlled environment with a temperature of 23°C ± 1°C and a 12/12‐h light–dark cycle. All animals were randomly divided into different experimental groups to ensure unbiased results, and the whole experimental procedures were collected and illustrated in Figure 1. The experiments were conducted in accordance with the Animal Ethics Committee of Wuhan University's Zhongnan Hospital (ZN2023265) and adhered to the National Institutes of Health Guidelines for the Care and Use of Laboratory Animals.

### 2.2 Drugs

There were several viruses involved in the current study, such as rAAV‐GfaABC1D‐hM3D(Gq)‐P2A‐mCherry (10 × 1012 V.G./mL), rAAV‐GfaABC1D‐hM4D(Gi)‐P2A‐mCherry(10 × 1012 V.G./mL), rAAV‐GfaABC1D‐mCherry (10 × 1012 V.G./mL), rAAV‐CaMKIIα‐hM3D(Gq)‐EGFP (10 × 1012 V.G./mL), rAAV‐CaMKIIα‐hM4D(Gi)‐EGFP (10 × 1012 V.G./ml), rAAV‐CaMKIIα‐EGFP (10 × 1012 V.G./mL), which were purchased from Shenzhen BrainCase Limited Company. Clozapine‐N‐oxide (CNO) (Tocris, 4936) were dissolved in dimethyl sulfoxide (DMSO) and subsequently diluted to their final concentrations (1 mg/kg) in sterile 0.9% saline.

### 2.3 Surgical Procedures

Surgical procedures were performed under ketamine anesthesia (dissolved in 1% pentobarbitone, intraperitoneal injection, 40 mg/kg, Jiangsu Hengrui Pharmaceuticals Co. Ltd., Lianyungang, China). It's worth mention that previous researches reported the rapid antidepressant effects only occurred in low dose ketamine [38, 39]. Studies suggest that only subanesthetic ketamine elicits antidepressant and anxiolytic effect [40]. However, in our experiments, we injected high dose ketamine, which only acted as an acute analgesic and the injected dose of each group was the same to counteract its other effect. Furthermore, the anxiety‐related effects of ketamine are scarce and controversial, our open field test (OFT) and elevated plus maze (EPM) tests were performed at least one‐week after injection of ketamine, the behavioral evalution could be almost unaffected. During the surgical procedures, mice were placed on a heating pad to maintain body temperature, which is crucial for ensuring physiological stability and facilitating recovery from anesthesia. Postsurgery, the mice were returned to their original cages and carefully monitored to ensure proper recovery.

#### 2.3.1 Neuropathic Pain Surgery

SNI surgery was conducted following the established protocol from a previous study [41]. Briefly, the fur on the left lateral thigh of the mice was shaved after administering general anesthesia, and a minor incision was made through the skin. The femoris muscle was carefully dissected to expose the sciatic nerve along with its three peripheral branches: the common peroneal nerve (CPN), the tibial nerve (TN), and the sural nerve. The CPN and TN were ligated using a 5–0 nylon suture and then cut 2 mm distal to the ligation to prevent nerve regeneration. It's important to protect the sural nerve to avoid causing paralysis. After completing the nerve ligation and sectioning, the incision was sutured using a 5–0 nylon suture. The sham‐operated mice were undergone the same surgical procedures, but the sciatic nerve was only exposed without ligation.

#### 2.3.2 Virus Injection

Mice were anesthetized and their heads were fixed in a stereotactic frame (RWD Life Science, Shenzhen, China). A small incision was made to expose the skull. The virus (150 nL/dosage) was bilaterally injected into ACC region (AP, +1.0 mm, ML, +0.3 mm, DV, −1.8 mm) at a rate of 100 nL/min using a glass pipette [42, 43]. Then the micro‐syringe was left in place for at least 10 min to prevent virus backflowing during the retraction. Following the injection, mice were placed on the heating pad for recovery, and then put them back in their original cages. At least 3 weeks later, mice were intraperitoneally administrated with 0.5 mg/kg CNO for pharmacogenetics studies before the undergoing behavioral experiments.

### 2.4 Animal Behavioral Assessments

The animal behavioral testing was performed under the same conditions to avoid any experimental bias. Before testing, all animals were habituated in the testing room with dim light (~20 lx) at least 1 h, and the software of ANY‐maze (Stoelting Inc., Kiel, WI, USA) was used to analyze their behaviors.

#### 2.4.1 Mechanical Allodynia Test

As previously described, the mechanical allodynia of the NP models was tested using a series of *von Frey* filaments (ranging from 0.008 g to 2.0 g) [10]. Mice were individually placed in acrylic compartments (7 × 9 × 7 cm^3^) with wire mesh pads at the bottom for at least 1 h of habituation until they calmed down. Pressure was then applied to the plantar surface of the left hind paw using the filaments. A positive response was defined as brisk paw withdrawal, flinching, or licking, indicating pain perception. Each test began with a 0.16 g filament; if a positive response occurred, a lower pressure filament was used next, and if a negative response occurred, a higher‐pressure filament was used. Each filament was applied five times per mouse. The up‐down method of Dixon was utilized to analyze the mechanical allodynia, providing a robust measure of pain sensitivity in the mice.

#### 2.4.2 Open Field Test

Before every testing, the apparatus (40 × 40 × 70 cm^3^) was cleaned with 75% ethanol. Mice were then placed in the apparatus to explore freely for 10 min. The time spent and the route taken in the center zone (20 × 20 cm^2^) were recorded and analyzed using ANY‐maze software.

#### 2.4.3 Elevated Plus Maze

The EPM apparatus consisted of two mutually perpendicular arms (74 × 5 cm^2^) raised 50 cm above the floor, with one closed arm having 14‐cm‐high walls and one open arm without walls. Before testing, each mouse was placed in the center zone facing an open arm and allowed to explore freely for 10 min. The time spent and the path taken in the open arm were recorded and analyzed using ANY‐maze software. After each test, the apparatus was thoroughly cleaned with 75% ethanol to eliminate any residual scents and ensure consistent conditions for subsequent tests.

### 2.5 Immunochemistry

To conduct immunohistochemical analysis on mouse brain tissue, mice were first subjected to deep anesthesia using 2% to 4% isoflurane before being transcardially perfused with phosphate‐buffered saline (PBS) to clear the blood from the vasculature. This was followed by perfusion with 4% paraformaldehyde (Merck, Darmstadt, Germany) to fix the tissues. Subsequently, the mice were decapitated, and their brains were carefully excised and immersed in 4% paraformaldehyde for 48 h to ensure thorough fixation. Following fixation, the brains underwent dehydration through immersion in 30% sucrose solution until they sank, indicating adequate dehydration. Then the dehydrated brains were frozen and sectioned into 40 μm‐thick coronal slices using a Thermo Fisher CRYOSTAR NX50 cryostat microtome (Thermo Fisher Scientific).

For immunostaining, the brain slices were washed three times for 10 min each in PBS to remove any residual fixative. Then, they were blocked for 2 h in solution containing 10% fetal bovine serum (FBS) with 0.3% Triton X‐100 in PBS to reduce nonspecific binding. The slices were incubated overnight at 4°C with primary antibodies against GFAP (goat‐anti‐GFAP, 1:500 dilution, ab53554, Abcam), NeuN (anti‐NeuN, 1:500 dilution, ab177487, Abcam), Iba‐1 (anti‐Iba‐1, 1:500 dilution, WX331590, ABclonal), CamKII (goat‐anti‐CamKII, 1:500 dilution, ab87597, Abcam), or c‐Fos (rabbit‐anti‐c‐Fos, 1:1000 dilution, 2250S, Cell Signaling Technology; rat‐anti‐c‐fos, 1:1000 dilution, 226,017, SYSY). After washing, the slices were incubated with species‐specific fluorescently labeled secondary antibodies (Alexa Fluor 488 Donkey Anti‐Goat IgG (H + L), 705–545‐003; Alexa Fluor 488 Donkey Anti‐Rabbit IgG (H + L), 711–545‐152; Cy3 Donkey Anti‐Goat IgG (H + L), 705–165‐003; Cy3 Donkey Anti‐Rabbit IgG (H + L), 711–165‐152; Goat anti‐Rabbit Alexa Fluor 647, 111–607‐008; Jackson ImmunoResearch; Alexa Fluor 488 Goat Anti‐Rat IgG (H + L), A11006, Thermo Fisher Scientific) for 2 h at room temperature. Finally, all slices were counterstained with 4′, 6‐diamidino‐2‐phenylindole (DAPI, C1002, Beyotime Biotechnology) for 10 min. The immunofluorescent images were captured using confocal microscope (Leica, TCS SP8, Buffalo Grove, IL) or a virtual microscopy slide‐scanning system (Olympus, VS. 120, Tokyo, Japan) for image acquisition. The images were further analyzed and processed using ImageJ software (National Institutes of Health, USA) to quantify and visualize the expression of the targeted proteins within the ACC region.

### 2.6 Western Blot

After various treatments, under the general anesthesia with isoflurane (2%–4%), mice brains were collected on an acrylic plate with ice in the bottom. Then, we harvested the ACC tissues and stored at −80°C. For protein extraction, ACC tissue is homogenized in RIPA buffer containing protease inhibitors (beyotime, P0013B), followed by centrifugation at 12,000 rpm for 15 min at 4°C to collect the supernatant for protein concentration measurement using a BCA assay (beyotime, P0012). Equal amounts of protein are then loaded onto SDS‐PAGE gels along with molecular weight markers (thermofisher, 26,617) and run at constant voltage. Proteins are transferred from the gel to a PVDF membrane. Then, membranes are blocked in 5% BSA (roche, G5001) in TBST for 2 h at room temperature, followed by overnight incubation at 4°C with primary antibodies against PSD95 (Abclonal, A0131) and GAPDH (Cell Signaling, 2118). After washing with TBST, membranes are incubated with HRP‐conjugated secondary antibodies (bioss, bs‐0295G) for 1 h at room temperature. Chemiluminescent substrate is applied to the membrane, which is then analyzed using a digital imaging system (Tanon‐4800). Finally, band intensities are quantified using image J software.

### 2.7 Statistics

All data were processed in GraphPad Prism v9.0 software, expressing as the mean ± standard error of mean (SEM). We used an unpaired *t*‐test for comparison between two groups, one‐way analysis of variance (ANOVA) followed by Tukey's post hoc test for comparison among ≥ 3 groups, two‐way ANOVA followed by unpaid *t* test for the comparisons of mechanical pain threshold between two groups, with statistical significance assessed as *p < 0.05, **p < 0.01, ***p < 0.001, ****p < 0.0001.

## 3 Results

### 3.1 SNI Induced Time‐Dependent Anxiety‐Like Behaviors and Correspondingly Overexcited ACC Astrocytes

**FIGURE 2:**
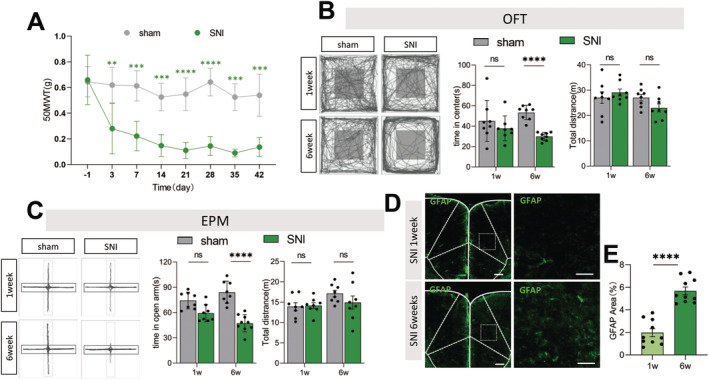
SNI mice manifested time‐dependent anxiety‐like behavior and correspondingly overexcited ACC astrocytes. (A) Time course of SNI‐induced the mechanical pain threshold, (*n* = 8 mice per group); Two‐way repeated‐measures ANOVA follow by unpaired *t* test; (B and C) The representative tracking plot, time in center zone (left) and total travel distance of for OFT (right) (B, *n* = 8 mice per group) and time in open arm (left) and total travel distance (right) for EPM test (C, *n* = 8 mice per group); Two‐way repeated‐measures ANOVA followed by post hoc Sidak's test; (D and E) Immunohistochemical staining and statistical data analysis of GFAP in the ACC after surgery (*n* = 10 brain slices from 5 mice); unpaired *t* test; Scale bar:100 μm. Ns *p* > 0.05, *p < 0.05, **p < 0.01, ***p < 0.001, ****p < 0.0001 sham versus SNI group.

The SNI‐induced NP model is a widely accepted chronic pain model that also presents with comorbid anxiety‐like symptoms. Following SNI surgery, SNI mice exhibited a significant reduction in pain threshold compared to sham‐operated mice, and this reduction persisted for 6 weeks (Figure 2A). To further investigate the anxiety‐like behavior in SNI mice, we conducted an OFT and found that, SNI mice at 6 weeks' postsurgery spent significantly less time in the center zone compared to their behavior at one‐week postsurgery (Figure 2B). Similarly, in the EPM test, SNI mice spent a shorter duration in the open arms at 6 weeks postsurgery compared to one‐week postsurgery (Figure 2C).

To determine whether astrocytes in the ACC were active in SNI mice with comorbid anxiety‐like behavior at 6 weeks, the ACC slices were immunostained for glial fibrillary acidic protein (GFAP), a marker of astrocytes. Results of immunostaining revealed a significant increase in GFAP expression in ACC of SNI mice at 6 weeks compared to one‐week postsurgery (Figure 2D,E). These findings suggested a temporal sensitivity in the development of anxiety‐like behavior following SNI surgery, with a corresponding increase in astrocyte activity in ACC.

### 3.2 Inhibited ACC Astrocytes Ameliorate Anxiety‐Like Behaviors in SNI Mice

**FIGURE 3:**
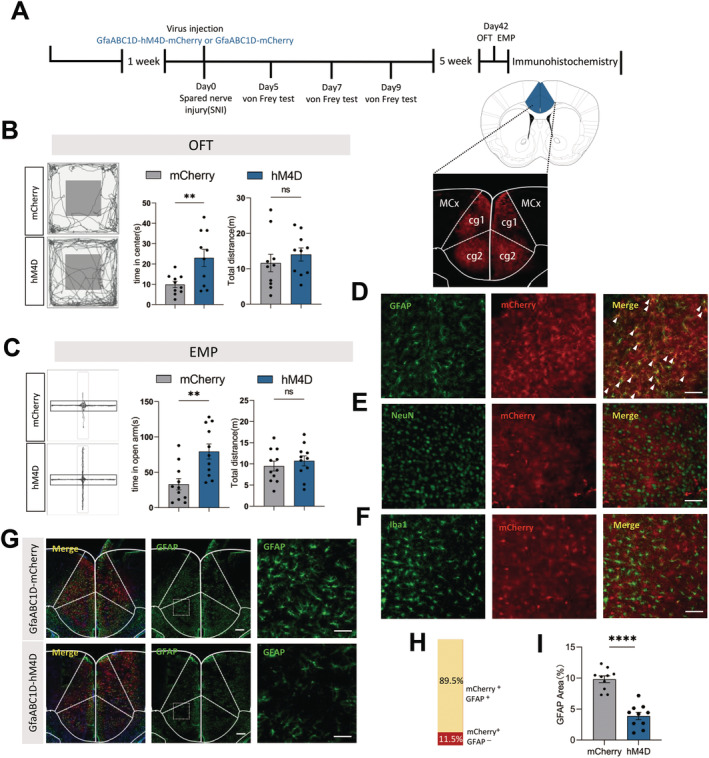
Inhibited ACC astrocytes ameliorate anxiety‐like behavior in SNI mice. (A) Timeline of the experimental procedures and a representative image illustrating gfaABC1D‐mCherry expression in the ACC; (B and C) The representative tracking plot, time in center zone (left) and total travel distance (right) for OFT (B, *n* = 11 mice per group) and time in open (left) and total distance (right) for EPM test (C, *n* = 11 mice per group); unpaired *t* test; (D–F) Immunohistochemical verification of hM4D expression in ACC astrocytes. The marker of astrocyte (D, GFAP, green), neuron (E, NeuN, green), microglial cell (F, Iba1, green) co‐immunostains with the GfaABC1D‐hM4D‐mCherry virus (red). Scale bar: 100 μm; (H) Histogram presenting the percentage of GFAP‐positive cells among hM4D‐positive cells. (*n* = 6 brain slice from 3 mice); (G and I) Immunohistochemical verification of GfaABC1D‐hM4D inhibition of astrocytes. (*n* = 15 brain slices from 5 mice); unpaired *t* test; Scale bar:100 μm. Ns *p* > 0.05, *p < 0.05, **p < 0.01, **p < 0.01, ****p < 0.0001 SNI 6 weeks + GfaABC1D‐mCherry versus SNI 6 weeks + GfaABC1D‐hM4D‐mCherry group.

To investigate the relationship of astrocyte activity and the anxiety‐like behaviors in SNI mice, the astrocytes in ACC were manipulated and their function were further evaluated. SNI surgery was performed concurrently with the virus injection (GfaABC1D‐hM4D‐mCherry), and the paw withdrawal threshold was evaluated on days −1, 5, 7, and 9 to screen for mice with mechanical allodynia (Figure S1A–C). Six weeks postsurgery, selected mice were injected intraperitoneally with 5 mg/kg CNO and subjected to behavioral tests 2 h later (Figure 3A). Our findings revealed that, following the chemogenetic inhibition of astrocytes, the mice in hM4D‐mCherry group spent significantly more time in the center zone of the OFT and in the open arm of the EPM test compared to mCherry control mice, while the total distances traveled showed no significant difference (Figure 3B,C). These results suggested that chemogenetic inhibition of ACC astrocytes could alleviate anxiety‐like behavior in SNI mice.

Six weeks postinjection, red fluorescence (~mCherry tag) was observed to be confined to the ACC region, confirming the virus infection (Figure 3A). The immunohistochemical staining revealed that most mCherry signals (about 90%) co‐localized with GFAP (Figure 3H), rather than NeuN or Iba‐1 (Figure S3A–D), indicating that the GfaABC1D‐hM4D‐mCherry virus specifically infected ACC astrocytes (Figure 3D–3F).

It is noteworthy that a study found that clozapine‐N‐oxide (CNO) could rapidly convert to clozapine in vivo, which could alter the mice behaviors [44]. To eliminate the potential confounding effects of CNO, a group of control animals were also injected with the GfaABC1D‐mCherry virus and compared. In comparison to the mCherry control group, CNO treatment could significantly inhibit ACC astrocytes in the hM4D‐mCherry group, demonstrating the virus's intended effect (Figure 3G,I).

### 3.3 Activated ACC Astrocytes Aggravate Anxiety‐Like Behavior and Regulate Excitatory Neurons

**FIGURE 4:**
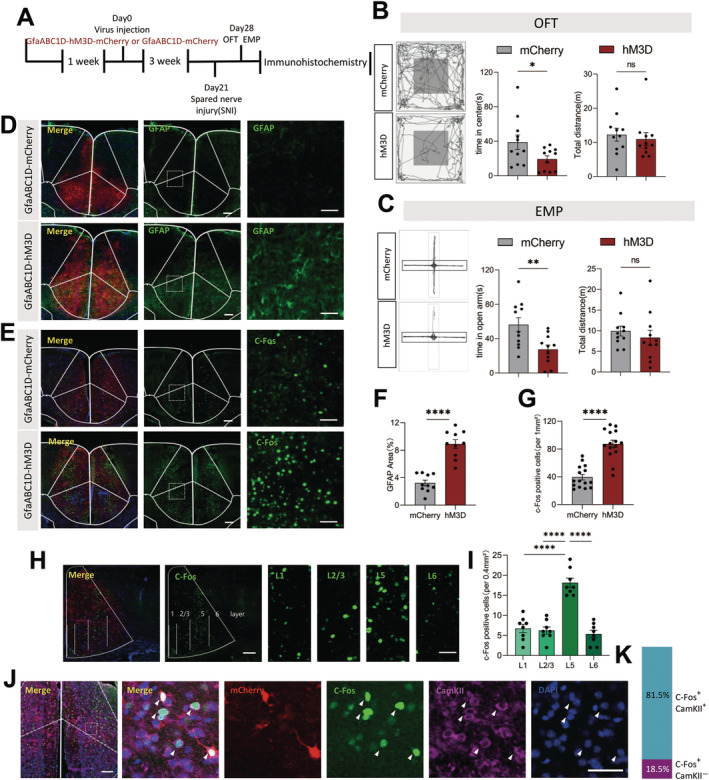
Activated ACC astrocytes aggravate anxiety‐like behavior and regulate excitatory neurons. (A) Timeline of the experimental procedures; (B and C) The representative tracking plot, time in center zone (left) and total travel distance (right) for OFT (B, *n* = 11 mice per group) and time in open arm (left) and total distance (right) for EPM test (C, *n* = 11 mice per group); unpaired *t* test; (D and F) Immunohistochemical verification of GfaABC1D‐hM3D activation of astrocytes (*n* = 15 brain slices from 5 mice); unpaired *t* test; Scale bar:100 μm; (E and G) Immunohistochemical staining and statistical data of c‐fos positive cells in the ACC (*n* = 15 brain slices from 5 mice); unpaired *t* test; Scale bar:100 μm; (H, I) Immunohistochemical staining and statistical data of c‐fos positive cells in individual layers in ACC (*n* = 15 brain slices from 5 mice); Two‐way repeated‐measures ANOVA follow by unpaired *t* test; Scale bar:100 μm (J) Histogram presenting the percentage of CamKII‐positive cells among c‐fos‐positive cells (*n* = 6 brain slice from 3 mice); (K) c‐fos colocalized with CamKII after GfaABC1D‐hM3D activation of astrocytes. Scale bar:100 μm. Ns *p* > 0.05, *p < 0.05, **p < 0.01, **p < 0.01, ****p < 0.0001 SNI 1 week + GfaABC1D‐mCherry versus SNI 1 week + GfaABC1D‐hM3D‐mCherry group.

Three weeks after virus injection (GfaABC1D‐hM3D), the treated mice were intraperitoneally injected with CNO, followed by a series of behavioral tests (Figure 4A). The naïve mice did not develop any anxiety‐like symptoms, indicating that the combination of GfaABC1D‐hM3D and CNO alone did not induce any anxiety‐like behaviors (Figure S2A,B). Furthermore, previous measurements indicated that mice did not exhibit anxiety‐like behaviors 1 week after SNI surgery, thus we try to proceeded with the animal behavior tests on the mice one‐week post‐SNI to investigate the roles of astrocytes in SNI. The mice were intraperitoneally injected with CNO, followed by OFT and EPM tests. These tests showed reduced time spent in the center zone and open arm zone, respectively, without affecting the total travel distance (Figure 4B,C). As expected, mice in the hM3D‐mCherry group developed anxiety‐like behaviors as early as one‐week post‐SNI. After completing the behavioral experiments, the mice were perfused 2 h after CNO treatment, and their brains were collected for staining. The GFAP density increased in hM3D‐mCherry mice, demonstrating that the virus effectively activated astrocytes (Figure 4D,F).

Previous studies have shown that the ACC excitatory neurons are overexcited in mice with anxiety‐like behaviors, and astrocytes are closely connected to neurons [45, 46]. To determine whether manipulating ACC astrocytes affected neurons, we activated astrocytes in hM3D‐mCherry mice and observed a significant enhancement in ACC *c*‐Fos expression, indicating that astrocytes influence the local neurons (Figure 4E,G). Besides, it is generally accepted that pyramidal cells in layer 5 project to the amygdala and periaqueductal gray, the former is a structure that has a key role in processing fear and anxiety, and the latter is involved in the descending modulation of spinal sensory transmission [11]. We investigated the tissue slices that attained from mice brains after activation of ACC astrocytes to count the number of c‐Fos positive cells. We found a significant increase in c‐Fos expression in the layer 5 compared with other layers in ACC (Figure 3H,I). And this result demonstrated that the function of ACC astrocytes in regulation of anxiety‐like behavior under the NP conditions should be through the regulation of layer 5 neurons. We then co‐stained layer 5 *c*‐Fos activated by astrocytes in hM3D‐mCherry mice with CaMKII, a marker of excitatory neurons, to detect the effects on local excitatory neurons. Most *c*‐Fos signals co‐localized with CaMKII (approximately 81.5%), suggesting that the majority of neurons activated by astrocytes were excitatory (Figure 4J,K). Overall, these results indicated that ACC astrocytes should play an important role in regulating the anxiety‐like behavior by modulating the L5 excitatory neurons.

### 3.4 Regulation of Anxiety‐Like Behaviors in SNI Mice by Manipulating ACC Excitatory Neurons

**FIGURE 5:**
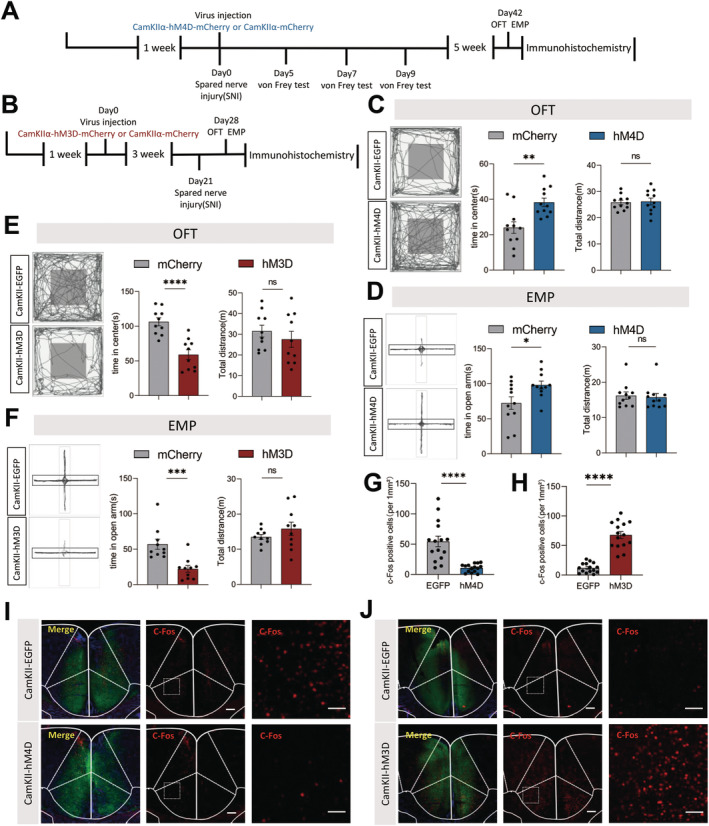
Chemogenetic manipulation ACC excitatory neurons can regulate anxiety‐like behavior in SNI mice. (A and B) Timeline of the experimental procedure; (C and E) The representative tracking plot, time in center zone (left) and total travel distance (right) for OFT after CamKII‐hM4D activation of astrocytes (C, *n* = 11 mice per group) or after CamKII‐hM3D activation of astrocytes (E, *n* = 10 mice per group); unpaired *t* test; (D and F) The representative tracking plot, time in center zone (left) and total travel distance (right) for EPM test after CamKII‐hM4D activation of astrocytes (D, *n* = 11 mice per group) or after CamKII‐hM3D activation of astrocytes (F, *n* = 10 mice per group); unpaired *t* test; (G–J) Immunohistochemical staining and statistical analysis of c‐fos positive cells in the ACC (*n* = 15 brain slices from 5 mice); unpaired *t* test; Scale bar:100 μm. Ns *p* > 0.05, *p < 0.05, **p < 0.01, **p < 0.01, ****p < 0.0001 SNI 6 weeks + CamKII‐mCherry versus SNI 6 weeks + CamKII‐hM4D‐mCherry group; SNI 1 week + CamKII‐mCherry versus SNI 1 week + CamKII‐hM3D‐mCherry group.

As showed above, ACC excitatory neurons were modulated by the local astrocytes. However, we needed to determine whether direct regulating the excitatory neurons would have the same effect on the anxiety‐like behaviors. To verify this, we injected CaMKII‐hM4D‐EGFP, CaMKII‐hM3D‐EGFP, or CaMKII‐EGFP viruses into the ACC of mice and waited at least 3 weeks for viral transduction (Figure 5A,B). Within the ACC, CaMKII‐hM4D‐EGFP expression was limited to NeuN, with small penetrance of GFAP or Iba1 (Figure S4A–D). Six weeks after SNI surgery, all mice were intraperitoneally injected with CNO. As expected, The CaMKII‐hM4D‐EGFP group mice spent more time in the center zone of the OFT test and the open arm of the EPM test, while the total distances they traveled were similar to the control group (Figure 5C,D). Conversely, after activating ACC excitatory neurons with CNO, the CaMKII‐hM3D‐EGFP group mice spent less time in the center zone and open arm compared to the animals in the CaMKII‐EGFP group (Figure 5E,F).

To further demonstrate that the CNO specifically inhibited or activated CaMKII‐hM4D‐mCherry or CaMKII‐hM3D‐mCherry respectively infected neurons, we examined the expression of *c*‐Fos in the ACC region. Following the activation of the CaMKII‐hM4D‐EGFP receptor by CNO, *c*‐Fos expression was scarcely detectable in the ACC (Figure 5G,I). In contrast, activation of ACC excitatory neurons led to a marked increase in *c*‐Fos expression (Figure 5H,J). These results indicated that modulating ACC excitatory neurons, much like the inhibition or activation of ACC astrocytes, significantly influences the anxiety‐like behaviors in SNI mice. In general, our results suggested that both astrocytes and excitatory neurons in the ACC played crucial roles in regulation of anxiety‐like behaviors under the NP conditions.

### 3.5 Activating Astrocytes Combined With Excitatory Neurons Inhibition in ACC Did Not Aggravate Anxiety‐Like Behavior

**FIGURE 6:**
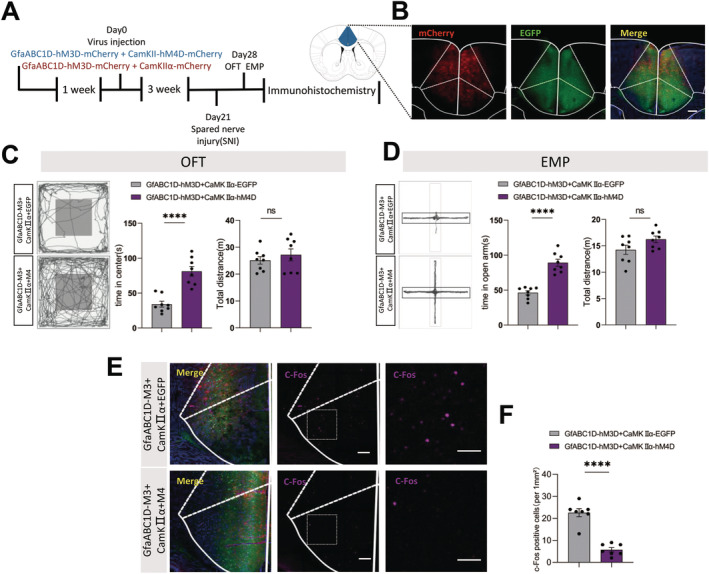
Activating astrocytes combined with excitatory neurons inhibition in ACC did not aggravate anxiety‐like behavior. (A) Timeline of the experimental procedures; (B) Representative image illustrating gfaABC1D‐mCherry and CamKII‐EGFP expression in the ACC; (C and D) The representative tracking plot, time in center zone (left) and total travel distance (right) for OFT (C, *n* = 8 mice per group) and time in the open arm (left) and total travel distance (right) for EPM test (D, *n* = 8 mice per group); (E and F) Immunohistochemical staining and statistical analysis of c‐fos positive cells in ACC (*n* = 6 brain slices from 3 mice). Scale bar: 100 μm. Ns *p* > 0.05, *p < 0.05, **p < 0.01, **p < 0.01, ****p < 0.0001 GfaABC1D‐hM3D‐mCherry+CamKII‐mCherry versus GfaABC1D‐hM3D‐mCherry+CamKII‐hM4D‐mCherry group.

To further investigate the combined effect of astrocytes and excitatory neurons in ACC, we developed two additional experimental groups: GfaABC1D‐hM3D‐mCherry + CaMKII‐hM4D‐EGFP, and GfaABC1D‐hM3D‐mCherry + CaMKII‐EGFP (Figure 6A). The viruses GfaABC1D‐hM3D‐mCherry, CaMKII‐hM4D‐EGFP, and CaMKII‐EGFP were all restricted to the ACC region (Figure 6B). Three weeks after virus injection, we conducted the behavioral tests. CNO led to decreased time spent in the center zone and open arm in the OFT and EPM tests, respectively, for mice in the GfaABC1D‐hM3D‐mCherry + CaMKII‐EGFP group compared to the GfaABC1D‐hM3D‐mCherry + CaMKII‐hM4D‐EGFP group (Figure 6C,D). To further investigate the combined effect of astrocytes and excitatory neurons, we examined the level of *c*‐Fos in ACC. The GfaABC1D‐hM3D‐mCherry + CaMKII‐hM4D‐EGFP group showed a significant increase in *c*‐Fos expression compared to the GfaABC1D‐hM3D‐mCherry + CaMKII‐EGFP group (Figure 6E,F). These results suggested that the activation of ACC astrocytes may regulate local excitatory neurons, contributing to the anxiety‐like behaviors in SNI mice.

### 3.6 Regulation of Synapse Density by Manipulating ACC Astrocytes

**FIGURE 7:**
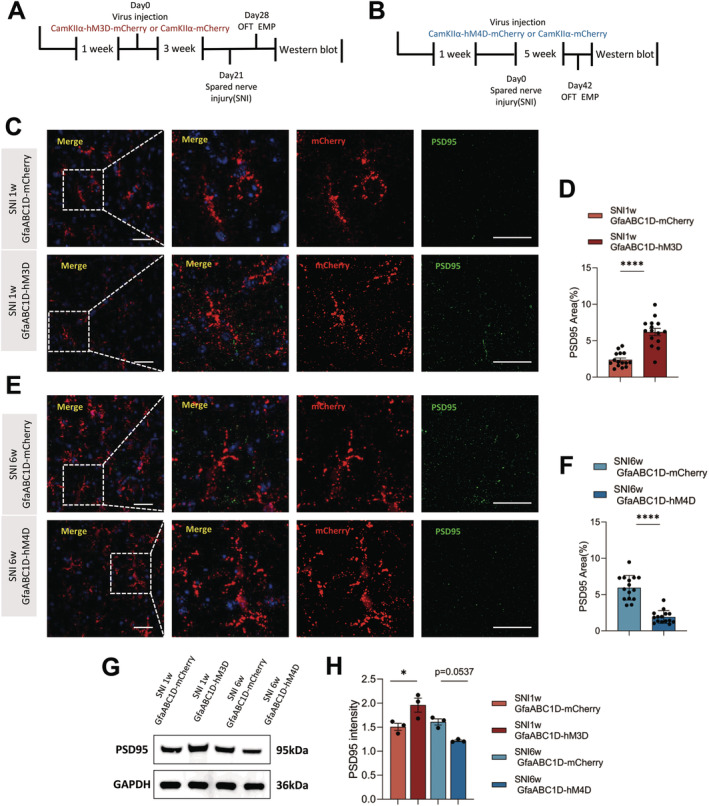
ACC astrocytes induced synapse‐related proteins. (A, B) Timeline of the experiment. (C–F) Representative fields (C, E) and relative quantification (D, F) of the PSD95 in the ACC L5 region. (*n* = 15 brain slices from 5 mice); unpaired *t* test; Scale bar: 50 μm. (G, H) Western blot analysis (G) and quantification (H) of in the whole ACC. (*n* = 3mice); Two‐way repeated‐measures ANOVA followed by unpaired *t* test. Ns *p* > 0.05, *p < 0.05, **p < 0.01, **p < 0.01, ****p < 0.0001 SNI 6 weeks + GfaABC1D‐mCherry versus SNI 6 weeks + GfaABC1D‐hM4D‐mCherry group, SNI 1 weeks + GfaABC1D‐mCherry versus SNI 1 weeks + GfaABC1D‐hM3D‐mCherry group.

In recent years, it has become clear that astrocytes play an important role in neuronal activity and plasticity [47]. To investigate whether synapse plasticity is involved in pain and consequent anxiety relief after the modulation of ACC astrocytes, we used GfaABC1D‐hM4D‐mCherry or GfaABC1D‐hM3D‐mCherry to inhibit or promote the ACC astrocyte activity, respectively, and then examined the effects of modulating ACC astrocyte activity on PSD 95 expression after behavior tests (Figure 7A). Immunofluorescence analysis revealed a lower density of the postsynaptic marker PSD95 in GfaABC1D‐hM4D‐mCherry mice in ACC L5 (Figure 7C,D). Conversely, activation of ACC astrocytes (GfaABC1D‐hM3D‐mCherry) resulted in higher PSD 95 expression compared to the GfaABC1D‐mCherry group (Figure 7E,F). Western blot of ACC homogenates also displayed a decreased expression of postsynaptic density protein (PSD95) in GfaABC1D‐hM4D‐mCherry mice and an increased expression in GfaABC1D‐hM3D‐mCherry mice (Figure 7G,H). In general, these results demonstrated that ACC astrocytes may induce increase synapse‐related proteins and modulate anxiety‐like behaviors.

## 4 Discussion

### 4.1 Main Findings

In this study, we addressed the knowledge gap concerning the contribution of ACC astrocytes to comorbid anxiety‐like behavior in the SNI model. During the course of post‐SNI, we found no significant differences in mice anxiety‐state and GFAP density in 1 week after surgery procedure while they manifest obvious decrease pain threshold. Mice would develop in anxiety and consequently increase the GFAP density when experiencing long‐term NP. Using immunohistochemical staining and western blot following chemogenetic manipulation of ACC astrocytes, we have demonstrated that activation of astrocytes in ACC could activate local excitatory neurons and increased synapse density. Additionally, our experiments revealed that manipulating the local excitatory neurons resulted in the same anxiety‐like behavior observed when manipulating astrocytes. These findings indicated a significant interplay between astrocytes and excitatory neurons in ACC, emphasizing that ACC astrocytes played a crucial role in the activation of excitatory neurons, and consequently, the development of anxiety‐like behavior in SNI mice. This study highlights the importance of targeting astrocyte activity in the ACC as a potential therapeutic strategy for managing anxiety in chronic pain conditions, providing a deeper understanding of the cellular mechanisms underlying these behaviors.

### 4.2 Anxiety Like Behaviors and Neuropathic Pain Animal Models

Among the NP animal models, SNI model has been extensively characterized, closely mimicking clinical NP symptoms [21]. Clinical and preclinical studies have demonstrated a strong association between NP and psychiatric conditions such as anxiety [48]. However, the published data regarding SNI's association with anxiety‐like behavior are contradictory. Some studies report no association between SNI and anxiety‐like actions [49], while others show a significant association between SNI and psychiatric disorders, including anxiety behavior [50, 51]. Our results suggested that mice developed psychiatric disorders in the later stages (at least 6 weeks postsurgery) and that this development was in a temporal fashion. Other studies on chronic pain comorbid with psychiatric disorders also show that experimental animals do not exhibit anxiety behavior 1 week after persistent pain stimuli. For instance, some studies reported that SNI mice displayed anxiety behavior after 2 weeks [8, 52], while others observed it after more than 4 weeks [53, 54]. This variability may result from different experimental conditions, such as mouse feeding, environment, and individual differences, but all studies agree that emotional disorders do not develop until the later stages postsurgery. Interestingly, we found that acute activation of astrocytes in naïve mice could not induce anxiety behavior. However, when activation occurred during the advanced stage of SNI, while anxiety behavior was not developing, mice manifested obvious anxiety disorders. Oher study also finds that in naïve mice without NP, acute ACC activation is not sufficient to trigger depressive‐like effects [3]. This suggests that the effect of astrocytes in the ACC on anxiety‐like behavior may be context‐dependent. Anxiety‐like behavior may be initially triggered by SNI surgery and subsequently promoted by the activation of astrocytes. These findings highlight the importance of timing and context in the development of anxiety‐like behaviors in NP models and underscore the crucial role of ACC astrocytes in modulating these behaviors.

### 4.3 Astrocyte Function for the NP‐Mediated Anxiety Animal Model

Historically, astrocytes were considered primarily as elements that maintain homeostasis and provide support for brain function. However, emerging evidence establishes that astrocytes act as active modulatory cells in synaptic function, neural circuits, and consequently on brain function and animal behavior [55]. Multiple studies over the past decade have reported astrocyte dysfunction in the brains of rodent models of chronic pain‐mediated anxiety. For instance, genetic or pharmacological inhibition of astrocytes in the ventral hippocampus (vHPC) compelety attenuated anxiodepressive‐like behaviors in chronic pain mouse model [56].;Beside, GFAP in the central nucleus of amygdala (CeA) were demonstrated to be increased at the chronic (4 weeks post‐SNL), while manipulating anxiety like behavior, but not acute (1 week post‐SNL), stage of NP [57]. Our research found that, in the context of NP and anxiety, astrocytes in the ACC play a pivotal role. Our results suggested that the activation of ACC astrocytes can exacerbate anxiety‐like behaviors, whereas their inhibition can ameliorate these behaviors. This modulation is mediated through the interaction between astrocytes and local excitatory neurons, highlighting the importance of astrocytic activity in regulating emotional responses associated with NP. Additionally, astrocyte activation is linked to other diseases, such as Alzheimer's disease [58], Parkinson's disease [59], Huntington's disease [59]. The role of astrocytes in comorbid anxiety behavior in these diseases warrants further investigation. Understanding the broader implications of astrocyte function could provide new insights into the treatment of anxiety across various neurological conditions.

### 4.4 Communications of Astrocytes and Neurons for the NP and Anxiety Animal Model

Accumulating evidence on astrocytes has established their role in bidirectional communication with neurons. Astrocytes respond to neurotransmitters and, in turn, release neuroactive substances that influence neuronal and synaptic activity [60, 61]. To further determine the relationship between astrocytes and neurons, we utilized SNI‐induced anxiety behavior mice and performed experiments on the interplay between astrocytes and excitatory neurons in the ACC in modulating anxiety‐like behavior. We found that activated astrocytes alone upregulated the c‐Fos level, whereas inhibited CaMKII neurons alone decreased it. However, the activation of astrocytes combined with the inhibition of neurons led to attenuated expression of c‐Fos and improved behaviors. This indicates that astrocytes may regulate local excitatory neurons by releasing neuroactive substances or through other molecular mechanisms in the development of anxiety‐like symptoms in the SNI model. By demonstrating the combined effects of manipulating both astrocytes and excitatory neurons, we provide evidence that these cell types work together to regulate anxiety‐like behaviors. Recent rodent research has demonstrated that CaMKII neurons are well correlated with anxiety behavior. For instance, adolescent cocaine exposure (ACE) mice exhibited anxiety‐like behavior accompanied by the activation of CaMKII‐positive neurons in the claustrum. By suppressing CaMKII activity, the anxiety behavior of ACE mice was reversed [62]. Similarly, in mice with plantar injection of complete Freund's adjuvant (CFA), activation of the dorsal raphe nucleus (DRN) GABAergic projections to ACC CaMKII neurons reversed comorbid anxiety behavior [63]. Additionally, administering a DREADD agonist (CNO) to selectively inhibit CaMKII neurons in the basolateral amygdala (BLA) significantly alleviated anxiety behavior induced by paclitaxel [64]. However, a recent study demonstrated that chemogenetic activation of CaMKII neurons in the ACC could robustly improve LPS‐induced behavioral deficits [65]. This contrast may be due to the different animal models used to induce anxiety behavior. This insight could inform future therapeutic strategies targeting both astrocytes and neurons to more effectively manage anxiety in chronic pain conditions.

### 4.5 Astrocyte‐Mediated Synapse Plasticity in NP and Anxiety Mouse Model

In general, Gq pathway activation of astrocytes is always considered depending on metabotropic glutamate receptor (mGluR)‐mediated intracellular calcium influx and tending to produce artificial phenotypes or mechanistic profiles [47]. Recent studies found that astrocytes also contributed to synaptic plasticity events and consequently accelerated chronic pain and anxiety‐like states [11, 47]. Astroglial processes enwrap most synaptic structures, forming the tripartite synapse [66]. Astrocytes become reactive in response to injury or disease in the nervous system, and reactive astrocytes were demonstrated to upregulate some genes responsible for the induction of synapse formation, which included unwanted synapses that lead to NP [67]. Initial studies on astrocyte‐mediated synapses plasticity in NP model focused on the spinal cord level, suggesting that reactive astrocytes release complex array of substances, such as gliotransmitters, chemokines, cytokines, and synaptogenic molecules to induce functional and structural synaptic plasticity [68]. Multiple lines of following evidences suggested maladaptive plastic changes in the “pain matrix” cortical regions, such as ACC, mPFC and primary somatosensory cortex also existed [68]. Besides, chronic restraint stress model with obvious anxiety‐like behavior displayed lasting dendritic hypertrophy, such as increased size of dendritic spine heads and the number of mature, mushroom‐shaped spines in basolateral amygdala (BLA) [69]. Similarly, these dendritic hypertrophy also existed in dorsomedial prefrontal cortex (dmPFC) and hippocampus after chronic stress [70]. On the other hand, LTP and other types of functional synaptic changes have been particularly well studied in the ACC, with reports on both presynaptic and postsynaptic contributions and increased AMPA receptor insertion [71]. By examining the synapse‐related proteins, such as PSD95, our results demonstrated that activation of astrocytes led to enhanced synaptic plasticity, which may increase inappropriate neural connections and result in aggravative anxiety‐like behavior in NP model.

## 5 Conclusions

In summary, our research emphasized the pivotal role of ACC astrocytes in modulating NP comorbid with anxiety behaviors. We found that the inhibition of ACC astrocytes could improve anxiety symptoms, while activation of astrocytes could induce these disorders in the advanced stage of SNI surgery. The modulatory effect of astrocytes on anxiety may occur by influencing the synapse plastic, which in turn affects the activity of excitatory neurons. This work highlights the importance of astrocyte dysfunction in the development of anxiety disorders associated with NP and underscores their potential as a target for antidepressant treatment. By targeting astrocytes, the current work may develop a more effective therapeutic strategy for managing anxiety in chronic pain conditions.

## Author Contributions

Qingqing Zhou: Conception and design, collection and assembly of data, data analysis and interpretation, and manuscript writing; Qi Zhong: Conception and design, data analysis, manuscript writing, and financial support; Zhuang Liu and Ziyue Zhao: Conception and design, collection and assembly of data, and data analysis; Jie Wang: conception and design, manuscript writing, and supervision; Zongze Zhang: manuscript writing, supervision, and financial support.

## Conflicts of Interest

The authors declare no conflicts of interest.

## Supporting information


Figures S1–S4.

## Data Availability

The data that support the findings of this study are available from the corresponding author upon reasonable request.
