# Supplementary material for: Modulating Anxiety‐Like Behaviors in Neuropathic Pain: Role of Anterior Cingulate Cortex Astrocytes Activation
Source: CNS Neurosci Ther. 2025 Jan 21;31(1):e70227. doi: 10.1111/cns.70227 (PMC11751476; doi:10.1111/cns.70227)
Supplement: Supplementary file 1 — Figures S1–S4. [file CNS-31-e70227-s001.doc]

**Supplement meterial：Modulating anxiety-like behaviors in neuropathic pain: Role of anterior cingulate cortex astrocytes**

**
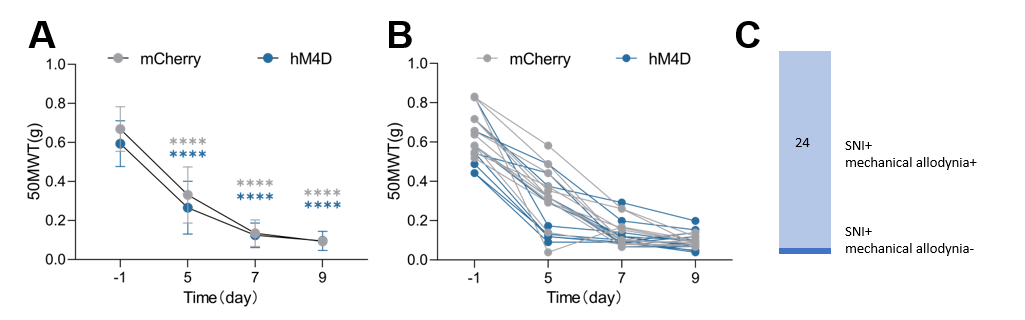
**

**Supplement Fig. 1. Most SNI mice manifested mechanical allodynia.**

(A and B) Time course of SNI-induced the mechanical pain threshold, (A, the mean threshold of per group; B, each mice threshold of per group, n=11 mice per group); (C) Histogram presenting the number of mice with or without mechanical allodynia among all SNI mice.(In the following experiments we only sorted the SNI mice with mechanical allodynia)

*Note: ns p>0.05, *p<0.05 ,**p<0.01, ***p<0.001, ****p<0.0001 d-1 vs d5, d7, d9*


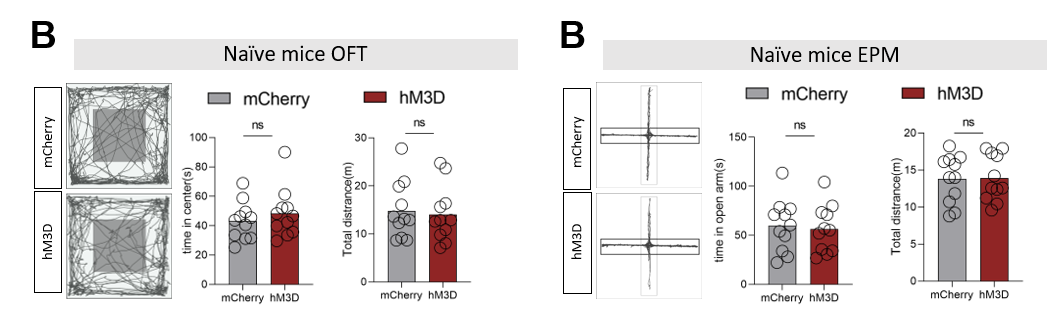


**Supplement Fig. 2. Activated ACC astrocytes in naïve mice did not aggravate anxiety-like behavior.**

(A and B) The representative tracking plot, time in center zone (left) and total travel distance of for OFT (right) (A, n=11 mice per group) and time in open arm (left) and total travel distance (right) for EPM test (B, n=11 mice per group).

*Note: ns p>0.05, *p<0.05 ,**p<0.01, ***p<0.001, ****p<0.0001 GfaABC1D-mCherry versus GfaABC1D-hM3D-mCherry group.*


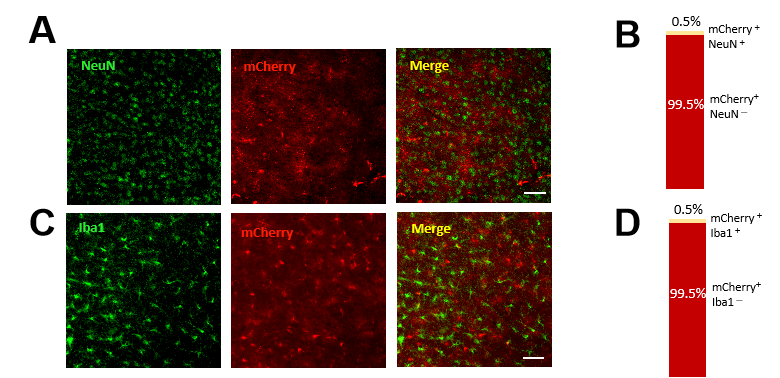


**Supplement Fig.3 Co-immunostains of hM4D‐mCherry and NeuN or IBa1.**

(A, C) The marker of neuron (A, NeuN, green) and microglial cell (C, Iba1, green) co-immunostains with the GfaABC1D-hM4D-mCherry virus (red). Scale bar: 100 μm; (B, D) Histogram presenting the percentage of NeuN‐positive cells and Iba1-positive cells among hM4D‐positive cells.(n=6 brain slice from 3 mice);


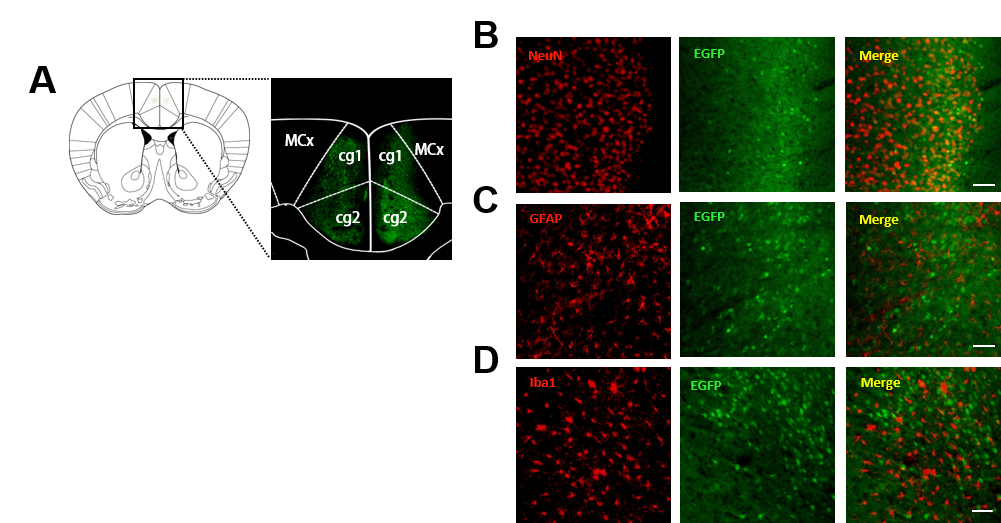


**Supplement Fig.4** **CamKII-EGFP expressed in the ACC.**

(A) Representative image illustrating CamKII-EGFP expression in the ACC; (B-D) Immunohistochemical verification of hM4D (green) expression in ACC neurons. The marker of neuron (B, NeuN, red), astrocyte (C, GFAP, red), microglial cell (D, Iba1, red) co-immunostains with the GfaABC1D-hM4D-mCherry virus (red). Scale bar: 100 μm;
